# Fulminant extraneural metastases in MYC-amplified Group 3 medulloblastoma: a pediatric case report

**DOI:** 10.1007/s00381-026-07351-4

**Published:** 2026-07-04

**Authors:** Lian Chen, Shaoqun Li, Lichao Wang, Hainan Li, Linbo Cai, Mingyao Lai

**Affiliations:** 1 Department of Oncology, Guangdong Sanjiu Brain Hospital, No. 578, Shatai South Road, Baiyun District, Guangzhou, 510510 Guangdong Province China; 2 Department of Pathology, Guangdong Sanjiu Brain Hospital, No. 578, Shatai South Road, Baiyun District, Guangzhou, 510510 Guangdong Province China

**Keywords:** Extraneural metastasis, Medulloblastoma, Molecular features, Prognostic factors

## Abstract

Medulloblastoma (MB) with *MYC* amplification is associated with aggressive clinical behavior and a high risk of metastatic dissemination. Extraneural metastasis (ENM), although rare in the modern treatment era, remains a devastating manifestation linked to poor survival. We report a pediatric case of MB with high-level *MYC* amplification and NanoString-based molecular subgrouping confirming Group 3 identity in a 9-year-old girl who developed rapidly progressive and widespread ENMs involving the bone marrow, liver, lymph nodes, and peritoneum shortly after craniospinal irradiation. Planned adjuvant chemotherapy could not be initiated because of persistent cytopenias associated with marrow infiltration during disease progression. Targeted next-generation sequencing identified somatic alterations in *PTEN*, *ARID2*, and *ERCC6*, as well as a heterozygous germline *SLX4* variant of uncertain clinical significance. While these findings do not establish a causal role in tumor progression, they further illustrate the molecular complexity of high-risk MB. The clinical course observed in this patient is consistent with prior reports linking *MYC* amplification to aggressive metastatic behavior. This case underscores the challenges of managing molecularly high-risk MB and highlights the importance of comprehensive molecular characterization and timely systemic therapy.

## Introduction

Medulloblastoma (MB) is one of the most common malignant pediatric brain tumors [1, 2]. Molecular classification identifies four principal subgroups, among which Group 3 is associated with the poorest prognosis, particularly in the setting of *MYC* amplification [3, 4]. Although MB commonly disseminates through cerebrospinal fluid pathways, extraneural metastasis (ENM) is rare in the contemporary treatment era, with a reported frequency of approximately 2%–5%, and is generally associated with dismal outcomes [5–8]. Bone, bone marrow, and lymph nodes are among the most frequently involved extraneural sites, whereas liver and peritoneal metastases are less common [5, 7]. We report a pediatric case of NanoString-confirmed Group 3 MB with high-level *MYC* amplification that developed rapidly progressive and widespread ENMs shortly after craniospinal irradiation (CSI).

## Case report

**Fig. 1:**
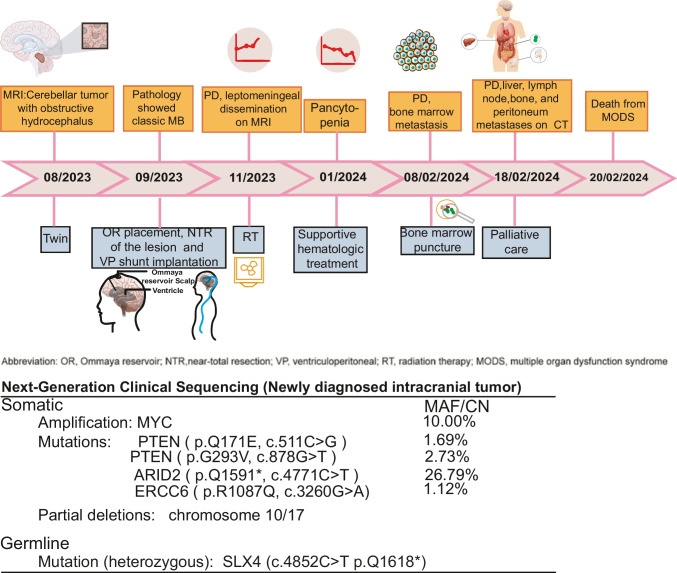
Timeline of the clinical history and NGS of the intracranial MB. MAF, mutation allele frequency; CN, copy number

**Fig. 2:**
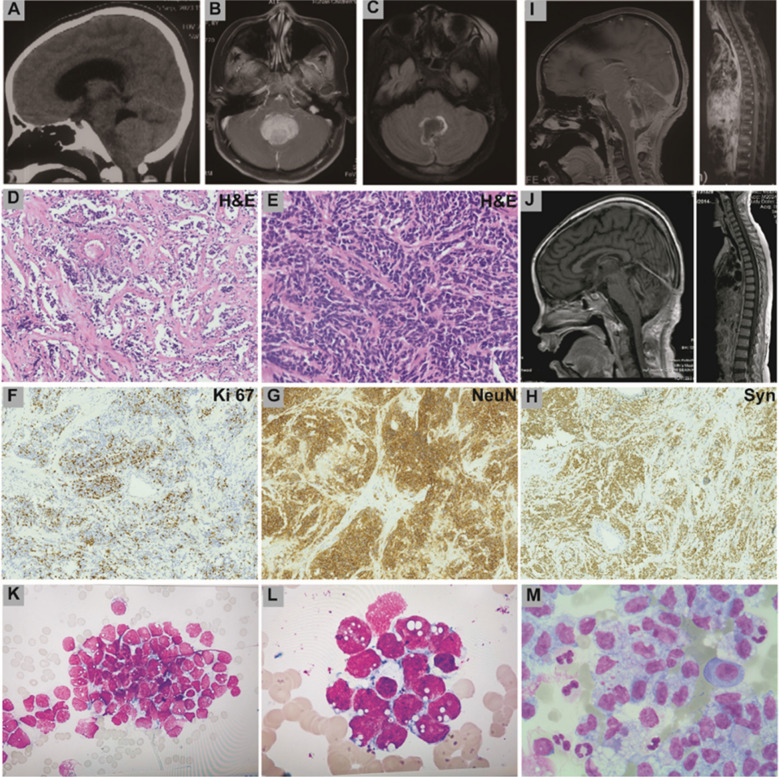
**A** Initial diagnostic head computed tomography (CT) scan of the patient with sagittal and (**B**) MRI T1 post-contrast axial image demonstrates a cerebellar lesion and obstructive hydrocephalus, **C** following near-total resection. **D**, **E** H&E staining (× 10, × 40) shows undifferentiated small cells in a sheet-like pattern with moderate nuclear pleomorphism. **F**–**H** Positive immunohistochemical staining of Ki-67, NeuN, and Syn. **I** MRI T1 post-contrast sagittal of the brain and spine image demonstrates cerebrospinal disseminated metastases **J** and following complete response after RT. **K**, **L** Bone marrow cytology showing malignant tumor infiltration. Because of limited sample material, additional immunohistochemical, FISH, or molecular confirmation was not feasible. **M** Tumor cells were found in the bloody ascites

Figure 1 provides an overview of the patient’s clinical course. A 9-year-old twin girl presented with a 1-month history of progressively worsening symptoms of hydrocephalus-related intracranial hypertension (such as headaches, dizziness, nausea, and vomiting) in August 2023. Subsequently, she developed ataxia (left-sided torticollis), prompting hospital admission on September 8, 2023. Brain magnetic resonance imaging (MRI) revealed a fourth ventricle mass with obstructive hydrocephalus (Fig. 2A, B). Due to sudden aphasia, an Ommaya reservoir was implanted, followed by near-total surgical resection of the lesion (Fig. 2C) and ventriculoperitoneal shunting.

Histopathological evaluation confirmed classic MB (Fig. 2D–H). Molecular subgroup classification using a NanoString-based gene expression assay demonstrated clustering within the Group 3 reference cohort. Targeted next-generation sequencing revealed high-level *MYC* amplification, somatic mutations in *PTEN*, *ARID2*, and *ERCC6*, and a heterozygous germline *SLX4* variant (c.4852C>T, p.Q1618*) (Fig. 1). Postoperatively, the patient developed cerebellar mutism syndrome, characterized by mutism, emotional lability, and ataxia. At the same time, considering the need for prolonged mechanical ventilation, a tracheotomy was performed. Her Karnofsky Performance Status was assessed at 40.

CSI was initiated on October 30, 2023. The patient’s consciousness worsened after 3.96 Gy CSI radiation therapy. Repeat MRI revealed leptomeningeal dissemination of MB (Fig. 2I). Hence, she underwent a 39.6 Gy CSI with a 50.4 Gy boost to posterior fossa and a 50.0–54.0 Gy boost to primary tumor bed. Post-radiation therapy MRI demonstrated near-complete response with only subtle enhancement surrounding the resection cavity (Fig. 2J). Three weeks after completion of radiotherapy, the patient developed rapidly progressive cytopenias.

**Fig. 3:**
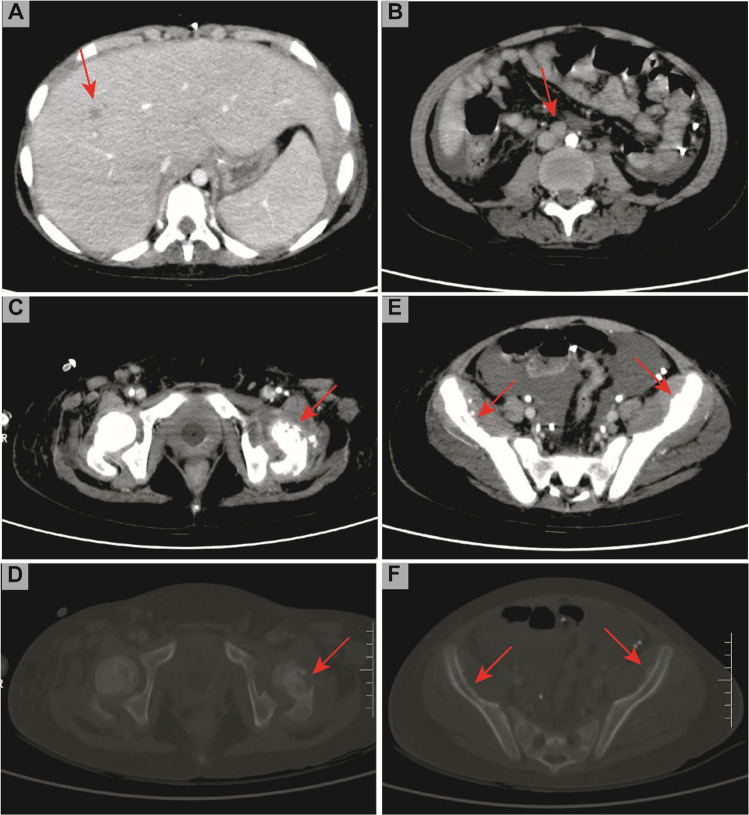
**A** Axial contrast-enhanced abdominal CT shows a low‐density liver lesion. **B** Retroperitoneal adenopathy. **C**, **D** Bone destruction in the left femoral neck. **E**, **F** Periosteal reaction in the bilateral iliac wings (arrows)

Subsequent bone marrow evaluation demonstrated malignant infiltration, which rendered the planned adjuvant chemotherapy infeasible. Because the amount of marrow material obtained was extremely limited in the setting of extensive marrow involvement, additional immunohistochemical, FISH, or molecular confirmation could not be performed. In the integrated clinicopathological context, and given that this event occurred during ongoing disease progression, the marrow lesion was considered most consistent with medulloblastoma metastasis (Fig. 2K, L). On February 12, 2024, she developed progressive jaundice, bilateral lower limb edema, and ascites. Laboratory tests revealed elevated transaminases, bilirubin, amylase, and lipase. Abdominal paracentesis yielded grossly bloody ascites with atypical tumor cells (Fig. 2M). Imaging revealed multiple hepatic lesions, retroperitoneal lymphadenopathy, and bone involvement in the iliac wings and left femoral neck (Fig. 3A–F). The patient transitioned to palliative care and passed away 1 week later.

## Discussion

This case illustrates an exceptionally aggressive clinical phenotype of pediatric MB characterized by NanoString-confirmed Group 3 identity, high-level *MYC* amplification, and fulminant multisystem extraneural dissemination. Although multimodal therapy has reduced the incidence of systemic dissemination, ENM continues to occur in biologically aggressive disease subsets. In the present case, NanoString-based subgrouping confirmed Group 3 MB, and high-level *MYC* amplification further supported an intrinsically high-risk tumor biology. *MYC*-driven Group 3 MB has consistently been associated with early relapse, rapid metastatic spread, and poor survival, and the fulminant clinical course observed in our patient is concordant with these established patterns [5, 9–11].

Notably, widespread systemic dissemination emerged shortly after completion of CSI and before initiation of the planned adjuvant chemotherapy. Early marrow involvement resulted in persistent cytopenias, rendering systemic therapy infeasible. Although direct immunohistochemical or molecular confirmation of the marrow lesion was not feasible because of limited sample material, the event occurred during ongoing frontline treatment and therefore represented disease progression rather than late relapse. It was interpreted in the context of documented medulloblastoma progression and subsequent widespread systemic dissemination. This sequence suggests that treatment limitation was a consequence, rather than a cause, of rapidly progressive disease. In selected high-risk tumors, fulminant systemic dissemination may therefore develop before standard systemic therapy can be effectively delivered. In addition, the ventriculoperitoneal shunt may have contributed to peritoneal dissemination [8], although it does not fully explain the simultaneous bone marrow, hepatic, nodal, and skeletal metastases observed in this patient.

Comprehensive genomic profiling identified additional somatic alterations in ***PTEN***, ***ARID2***, and ***ERCC6***, as well as a heterozygous germline ***SLX4*** variant. Public database review did not identify the same ***SLX4*** variant in ClinVar or cBioPortal. Therefore, the clinical significance of this specific heterozygous variant in medulloblastoma remains uncertain. In addition, chromosomal breakage sensitivity testing was not performed on peripheral blood or bone marrow cells; thus, an underlying constitutional DNA repair deficiency could not be further evaluated. Accordingly, this finding should be regarded as a variant of uncertain significance rather than a proven driver of tumor progression or metastasis.

The biological mechanisms underlying ENM in MB remain incompletely understood. Prior studies have suggested that molecular subgroup, *MYC* family amplification, treatment exposure, and disruption of anatomical barriers may collectively influence metastatic patterns [5]. However, because ENM is rare, current evidence is derived largely from isolated case reports and small case series. Larger molecularly annotated cohorts will be needed to clarify the determinants of systemic dissemination and to better define patients at highest risk.

From a therapeutic perspective, *MYC*-amplified Group 3 MB remains an area of major unmet clinical need. Early molecular risk stratification, including subgroup confirmation and genomic profiling, may support timely therapeutic planning and consideration of intensified or alternative treatment strategies in selected patients. In cases characterized by early systemic dissemination or marrow involvement, prompt recognition of aggressive disease biology is critical for both supportive care and treatment decision-making.

## Conclusion

This case highlights the fulminant clinical trajectory that can occur in molecularly high-risk MB and the practical difficulty of delivering systemic therapy in the setting of rapid disease progression. Molecular subgroup validation using NanoString-based profiling, together with genomic characterization, provides important biological context but does not, in isolation, establish causal mechanisms of metastasis. As a single-case observation, this report cannot determine causality; nevertheless, it expands the clinicomolecular spectrum of aggressive MB with extraneural dissemination.

## Data Availability

This data is available upon reasonable request.

## Abbreviations

*CSI*: Craniospinal irradiation
*ENM*: Extraneural metastasis
*MB*: Medulloblastoma
*MRI*: Magnetic resonance imaging
